# Cultivation of *Chlorella sorokiniana* IPPAS C-1 in Flat-Panel Photobioreactors: From a Laboratory to a Pilot Scale

**DOI:** 10.3390/life12091309

**Published:** 2022-08-25

**Authors:** David A. Gabrielyan, Maria A. Sinetova, Boris V. Gabel, Alexander K. Gabrielian, Alexandra G. Markelova, Margarita V. Rodionova, Vladimir S. Bedbenov, Natalia V. Shcherbakova, Dmitry A. Los

**Affiliations:** K.A. Timiryazev Institute of Plant Physiology, Russian Academy of Sciences, Botanicheskaya Street 35, 127276 Moscow, Russia

**Keywords:** *Chlorella*, biomass, cultivation, flat-panel photobioreactor, microalgae

## Abstract

Flat-panel photobioreactors are effective systems for microalgae cultivation. This paper presents the growth characteristics of the microalgae *Chlorella sorokiniana* IPPAS C-1 as a result of three-stage scale-up cultivation in a specially designed cultivation system. First, *C. sorokiniana* was grown aseptically in 250 mL glass vessels; then, it was diluted and inoculated into a 5-liter flat-panel horizontal photobioreactor; and, at the last stage, the culture was diluted and inoculated into a 70-liter flat-panel vertical photobioreactor. In the presented cycle, the cultured biomass increased by 326 times in 13 days (from 0.6 to 195.6 g dw), with a final biomass concentration of 2.8 g dw L^−1^. The modes of semi-continuous cultivation were considered. The biomass harvest and dilution of the suspension were carried out either every day or every 3–4 days. For *C. sorokiniana* IPPAS C-1, a conversion coefficient of optical density values to dry biomass (g L^−1^) was refined through a factor of 0.33. The key parameters of the photobioreactors tested in this work are discussed.

## 1. Introduction

Microalgae and cyanobacteria are photoautotrophic organisms that can use inorganic carbon and light energy to produce organic matter via photosynthesis. They have higher growth rates and photosynthesis efficiency compared to higher plants [1,2]. Unicellular algae have been the subject of mass cultivation since the 1940s of 20th century, initially for the purpose of human nutrition and protein storage. Later, algal biomass was considered as a source for animal foods, food supplements, and pharmaceuticals [3,4,5]. Nowadays, microalgae are widely applied for wastewater treatment [6], CO_2_ utilization [7], and the production of biofuels [8,9].

Microalgae may be grown in outdoor open ponds under sunlight in uncontrolled conditions. Alternatively, they may be cultivated indoors in large-scale photobioreactors (PBRs) that allow for the production of high-quality and -purity biomass under controlled conditions of illumination, temperature, pH, etc. [10]. Twenty years ago, nearly all the commercial production of microalgae was based on open pond technologies. However, nowadays, the annual world-wide production of the commonly used *Chlorella* and *Arthrospira*/*Spirulina* biomass (~20,000 tons) is equally distributed between open reservoirs or PBRs that employ sunlight and atmospheric CO_2_ and PBRs that produce microalgae either photoautotrophycally—with artificial illumination and compulsory purge with a gas-air mixture (GAM)—or heterotrophically—in the dark using sugars as a source of carbon and energy [10].

Although the PBR-based production of microalgae provides several advantages such as minimizing toxicity and ensuring the stable conditions of cultivation, the designing and scaling-up of PBRs still remain a challenge. The basic problem is that the ability of microalgae to utilize light quanta as an energy source is limited by high population densities. The large energy density at the boundary surface contrasts with the insufficient supply to the cells at only a few mm of layer thickness [11].

Examples of the PBRs include bubble and airlift columns, stirred tanks, helical tubes, and conical, torus, tubular, and flat-panel PBRs [12]. Various modifications of flat-panel PBRs usually have a higher photosynthetic light conversion efficiency than other types of PBRs [12,13]. Moreover, flat-panel PBRs benefit in space requirements compared to other cultivation systems. While the productivity of tubular-type PBRs varies from 0.1 to 1.0 g of dry weight per liter per day, flat-type PBRs provide up to 5 g dw L^−1^ d^−1^ depending on the cultured strain and the construction features of the bioreactor [11,12,13,14,15].

The final productivity in terms of biomass accumulation also depends on the choice of the cultivation technology—batch or continuous—which, in turn, is determined by the tasks, environmental conditions, and other objective and/or subjective reasons. In the batch mode, all nutrients are added at the beginning of the cultivation, cultures grow in controlled conditions (temperature, light, CO_2_ concentration) with no other supplements. Batch culture passes over several growth stages: lag, accelerating, exponential (logarithmic), decelerating, stationary, and inhibitory (death) phases. At the stationary stage, a maximum density of a microalgae suspension is reached, and the cultivation may be stopped: a PBR is then emptied and cleaned, and the cycle is repeated. This mode may take from several days to several weeks with the periodic harvesting and cleaning of the reactor [14,15].

The continuous mode (chemostatic or turbidostatic cultivation) allows for the periodical withdrawal of certain volumes of cell suspension. The chemostat method is often used in the microbiological industry. It maintains the rates between the biomass harvesting and the supplementation of a culture with a fresh medium. The turbidostatic method implies the maintenance of a steady-state biomass level by a continuous dilution of the cell suspension with a nutrient medium, which allows for the constant absorption of light energy by cells. The latter method is more advanced and preferable for the cultivation of microalgae; however, it requires additional costs for special measuring and control systems.

In industrial cultivation, special attention is paid to the semi-continuous mode: when a certain density value is reached at the linear stage of growth, a part of the suspension is harvested, and the biomass is separated from the nutrient medium. Then, either the used medium supplemented with required nutrients is returned to a PBR or the fresh medium is added to reach the initial volume. Periodically, total refreshments of the culture medium are also useful to avoid the critical accumulation of algal metabolites in the suspension [14,15].

Here, we present an algal cultivation system based on semi-closed PBRs, which can be applied for various scientific and biotechnological purposes. We describe the design features of these PBRs and the parameters of the biomass growth for the microalgae *Chlorella sorokiniana* IPPAS C-1, starting from a glass vessel with an inoculum of 0.25–0.3 L and finishing with a pilot industrial volume of 70 L in both the batch and semi-continuous modes, which resulted in an increase in biomass from 0.6 to 195.6 g dw within 13 days of cultivation.

## 2. Materials and Methods

### 2.1. Microalgae Strain and Maintenance Conditions

The axenic strain of *Chlorella sorokiniana* IPPAS C-1 was obtained from the collection of microalgae and cyanobacteria IPPAS (K.A. Timiryazev Institute of Plant Physiology, RAS, Moscow, Russia). The axenic culture was maintained on slants of a Tamiya-agarized medium [16] in glass tubes at 22 °C under continuous illumination with cool white luminescent lamps of 30 μmol photons m^−2^ s^−1^. For the experiments, cells of *C. sorokiniana* were grown for 10–14 days in 300 mL Erlenmeyer flasks with 100 mL of ½ Tamiya-modified medium on an orbital shaker at room temperature under an average illumination of 50 μmol photons m^−2^ s^−1^ from warm white light-emitting diodes (LEDs). The ½ Tamiya-modified medium composition, g L^−1^: NaNO_3_—2.1; MgSO_4_ × 7H2O—1.25; KH_2_PO_4_—0.625; FeSO_4_ × 7H_2_O—0.009; EDTA—0.037; trace element solution (TES) 1 mL L^−1^; TES composition, g L^−1^: H_3_BO_3_—2.86; MnCl_2_ × 4H_2_O—1.81; ZnSO_4_·7H_2_O—0.222, MoO_3_ × 2H_2_O—0.018, NH_4_VO_3_—0.023. All measurements of the irradiation level in the working volume of PBRs and on the surface of the LED modules were recorded with a quantum meter LI-189 with a LI-190SA quantum sensor (LI-COR, Lincoln, NE, USA) and expressed in μmol photons m^−2^ s^−1^.

### 2.2. Algal Pre-Culture for PBR Inoculation

The initial culture of *C. sorokiniana* IPPAS C-1 was grown aseptically in a glass vessel with 250 mL of ½ Tamiya medium for 2–3 days at 32 ± 0.6 °C under continuous illumination of 500 μmol photons m^−2^ s^−1^ provided by warm white, blue, and red LEDs. Culture mixing and aeration were achieved by bubbling with a sterile gas-air mixture (GAM) that contained 1.5–2% CO_2_. Pure CO_2_ from a pressured gas cylinder was mixed with air by a compressor in a mixing tank. The GAM passed through filters, rotameters, a CO_2_ concentration detector, and a humidifier and eventually entered into the cell suspension through sprayers. In PBR FPH-5, the volumetric flow rate of the supplied mixture was 2.09 ± 0.26 L min^−1^, while in PBR FPV-70, it was 8.79 ± 1.21 L min^−1^. The CO_2_ concentrations in the GAM were estimated with the gas analyzer PKU-4 N-M-T (JSC “ES&S”, Moscow, Russia), with an absolute error limit of ±(0.1 + 0.05·C), where C is the nominal value of CO_2_ concentration. The exponentially growing culture was diluted with a fresh medium; two 250 mL aliquots were transferred to new glass vessels and grown for another 3 days at similar conditions. The resulting culture with a dry biomass concentration of 1.65–4.29 g dw L^−1^ was used as the inoculum for the next stage of cultivation. The laboratory cultivation system—which holds 250 mL glass vessels and controls three main cultivation parameters: temperature, light intensity, and CO_2_ supplementation—is presented in Supplementary Figure S1 and Video S1 and will be described in detail elsewhere.

### 2.3. Flat-Panel 5 L Horizontal PBR (FPH-5)

**Figure 1:**
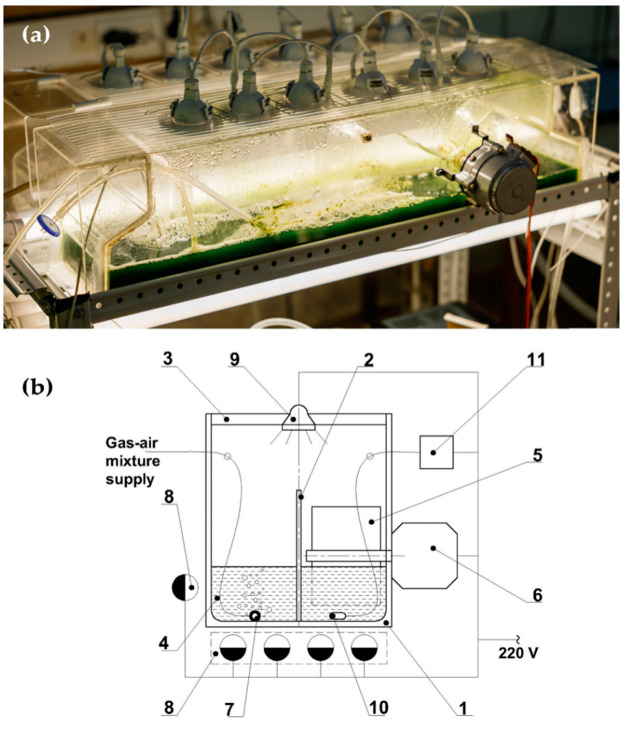
Flat-panel horizontal photobioreactor FPH-5: Photograph (**a**) and scheme (**b**) of the main PBR units. 1—PBR reservoir; 2—Partition wall; 3—Lid; 4—Suspension; 5—Impeller; 6—Electric motor; 7—Bubbling tube; 8—Lower LED Module; 9—Upper LED module; 10—Temperature sensor; 11—Temperature indicator.

The cultivation of the inoculum was carried out in two PBR FPH-5s with a nominal volume of 5 L each (Figure 1). The PBR FPH-5 was made of parallelepiped Plexiglas^®^ with dimensions of 75 × 16 × 19 cm. Lighting was provided from the top and from the bottom of the reactor. The top system consisted of six circular LED lamps (In HOME LED-JCDR-VC 11W, 820 lm, and 3000 K) that were installed in the reactor lid and illuminated the culture from the top (Figure 1b, position 9). The bottom system consisted of five cylindrical LED lamps (FL-LED T8-900-15W, 1500 lm, 3000 K) that were installed in a platform: four lamps under the PBR and one on the side (Figure 1b, position 8). The maximum irradiation on the suspension surface from the top illumination system reached 480 μmol photons m^−2^ s^−1^ when all six lamps were turned on. The bottom illumination system provided up to 253 μmol photons m^−2^ s^−1^.

The stirring of the culture was provided by low-speed motors (1–1.5 rpm) installed on the side of each PBR rotating a shaft with blades (Figure 1b, positions 5 and 6). The impeller blades defined the flow direction and provided the mixing of the suspension layers. In PBR channels, the flow was limited by the side walls of the reactor and by the partition wall in the middle of it. A perforated plastic tube was installed on the side opposite from the motor and provided the GAM supply in the form of small bubbles. GAM passed through polytetrafluoroethylene filters (Millex-FH Filer Syringe Filter Unit, 0.45 μm) and through a humidifier before entering into the PBR. The filling of the PBR with the inoculum and culture medium, as well as the draining of the suspension, is provided through the tap at the bottom at the end part of the PBR. The reactor was equipped with a temperature sensor installed on the bottom of the reactor through the hole in the upper part of the reservoir. Suspension heating is provided by infra-red irradiation from a lighting system. In case of overheating the PBR is cooled by an external fan. The operation of PBR FPH-5 is demonstrated in Video S2.

For the second stage of the cultivation, 250 mL of the culture from the previous stage was diluted with ½ Tamiya medium up to concentration of 0.08–0.15 g dw L^−1^ The experiments were carried out in two modes: batch (3–5 days) and semi-continuous (with a period of 1 or 3 days) modes. The culture was grown at 30–32 °C for the batch mode and at 32–36 °C for the semi-continuous mode under the continuous irradiation of 250 μmol photons m^−2^ s^−1^ from each side (average irradiation: 500 μmol photons m^−2^ s^−1^). The light path inside the suspension was 50 mm. The concentration of CO_2_ in the GAM was 1.5–2%. The culture with a final concentration of 2.64–3.3 g dw L^−1^ was used for the inoculation into the flat-panel vertical PBR with a working volume of 70 L (FPV-70).

The water for all PBRs was purified by the six-stage reverse osmosis system AP-800DIR-400 (AquaPro Industrial Co., Ltd., Nanking E. Rd., Taipei, Taiwan). All containers, hoses, filters, and liquids were either sterilized by autoclaving or treated with hot steam and 70% ethanol.

### 2.4. Flat-Panel 70 L Vertical PBR (FPV-70)

**Figure 2:**
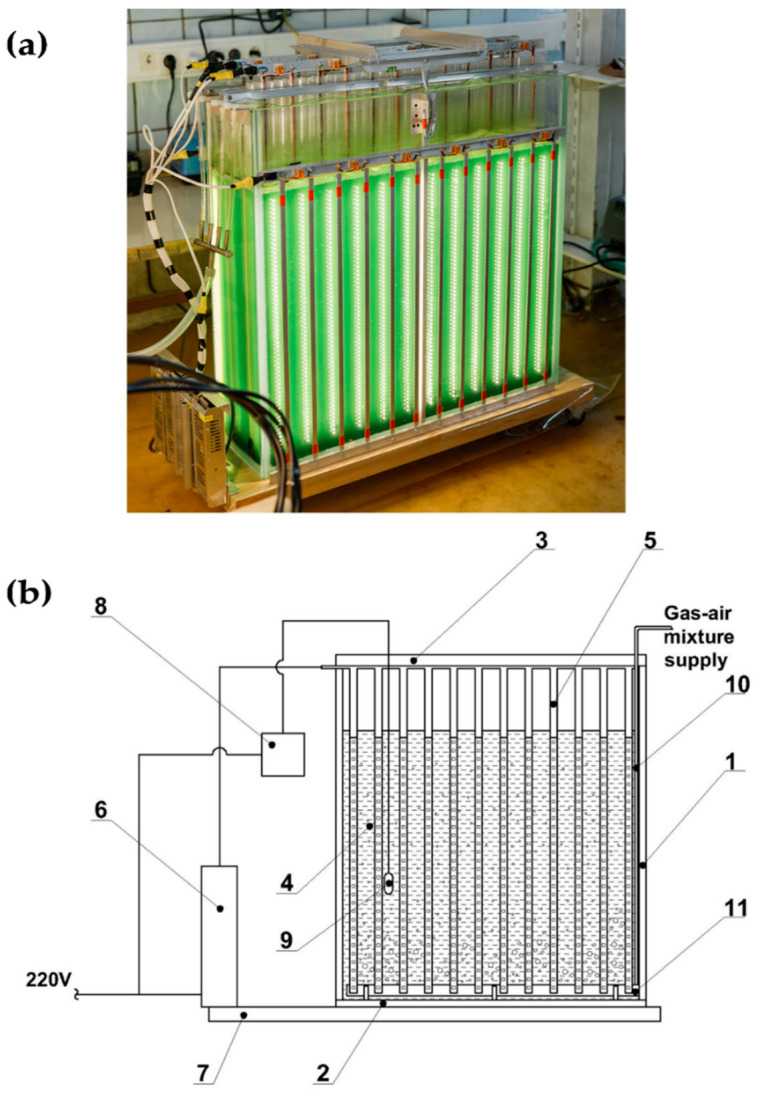
Flat-panel vertical photobioreactor FPV-70: Photograph (**a**) and general scheme (**b**) of the main PBR units. 1—PBR reservoir; 2—PBR bottom; 3—PBR Lid; 4—Suspension; 5—LED module; 6—LED module power supply; 7—Platform; 8—Temperature indicator; 9—Temperature sensor; 10—GAM supply tube; 11—GAM sprayer.

The PBR FPV was designed to solve two main problems of microalgae cultivation: (a) the additional illumination of thin layers of algal suspension with high densities [11,14,17]; and (b) the increase in the path length of the GAM for increasing the amount of CO_2_ fixed by the culture [18,19]. The PBR FPV-70 was a rectangular glass aquarium with dimensions of 750 × 710 × 22 mm and a wall thickness of 10 mm (Figure 2). The lighting system was made of three LED modules immersed into the reactor and two LED modules located at the outer side walls. The LED modules consisted of LED strip lights (9.6 W m^−1^-SMD2835-120 LED-Warm (3000 K)-12V-IP20) installed in the channels of cellular polycarbonate sealed from the bottom. The modules were independent from each other and could be removed/inserted from/into the PBR one by one. Strip lights were placed on aluminum strips to remove the heat generated during their long-term operation. The average irradiation on the surface of each LED was 1060 μmol photons m^−2^ s^−1^.

The mixing of the culture and the GAM supply was carried out using aquarium spray hoses installed between the submersible LED modules at the bottom of the PBR. A temperature sensor was also installed at the bottom of the PBR. Suspension heating was provided by infra-red irradiation from the lighting system. In the case of overheating, the PBR was cooled by an external fan. The operation of PBR FPV-70 is demonstrated in Video S3.

For the final stage of the cultivation, the culture from the previous stage was diluted with ½ Tamiya-modified medium to a concentration of 0.17–0.27 g dw L^−1^. The culture was grown at 30–38 °C. The light path inside the suspension was 50 mm. In the experiments, two submersible and two external LED modules were used. The average irradiation of the external light-receiving PBR surfaces was 21 μmol photons m^−2^ s^−1^, and that of the inner surfaces was 122 μmol photons m^−2^ s^−1^ (the average irradiation was 143 μmol photons m^−2^ s^−1^). The light path lengths inside the suspension from the external LED module to the submersible ones, between the submersible LED modules, and between the submersible and the external LED modules were 50, 100, and 50 mm, respectively. The concentrations of CO_2_ in the GAM were 0.5–1%, 1.5–3%, 3–6%, and 3–9%. The experiments were carried out in two variants: batch (5–9 days) and semi-continuous (with a period of 1 or 3–4 days) modes. The final dry biomass concentration in the suspension was 2.8 g dw L^−1^ in the batch mode.

### 2.5. Growth Characteristics

The growth rate was estimated by changes in the OD of suspension and by the dry weight. The OD was measured at 750 nm by the Genesys 10S UV-V spectrophotometer (Thermo Scientific, Waltham, MA, USA). To measure the dry weight, 1–10 mL samples were precipitated by centrifugation, washed with distilled water, and dried at 80 °C for 12 h in pre-weighed plastic microtubes in a heat oven. The dry weight of each probe was measured in triplicate.

The following parameters were used to estimate the growth rate.

- Conversion coefficient (k) between OD_750_ and biomass concentration:
  (1) $$k=\;\rho /{\mathrm{OD}}_{750}\;$$

OD measured at 750 nm is primarily dependent on light scattering and is thus proportional to the biomass concentration (ρ). Parameter k may be defined empirically for each particular organism and each particular spectrophotometer [20]. In this work, five probes were analyzed from (a) two independent experiments from PBR FPH-5; (b) two experiments from PBR FPV-70; and (c) one experiment from 250 mL glass vessels incubated in the laboratory cultivation system. Both the OD_750_ and dry biomass concentration were estimated in each individual probe.

- The specific productivity for the batch mode (P_sp_) was estimated by dry weight (g dw L^−1^d^−1^):
  (2) $$P_{\mathrm{sp}}=\left(\rho_{2}-\rho_{1}\right)/\left(t_{2}-t_{1}\right)$$
  where ρ_1_ and ρ_2_ are the biomass concentrations measured at time 1 (t_1_) and time 2 (t_2_).

Using the conversion coefficient k between the OD_750_ and biomass concentration, the following equation was applied:

(3) $$P_{\mathrm{sp}}=k\cdot \left(\mathrm{OD}750_{2}-\mathrm{OD}750_{1}\right)/\left(t_{2}-t_{1}\right)$$

where OD750_1_ and OD750_2_ are the optical densities of the culture at 750 nm measured at time 1 (t_1_) and time 2 (t_2_)

- The specific productivity for the semi-continuous mode ($P_{\mathrm{sp}}^{\prime }$) was also estimated by dry weight (g dw L^−1^ d^−1^):
  (4) $$P_{\mathrm{sp}}^{\prime }=\frac{M_{\mathrm{total}}-M_{0}}{t\cdot V_{\mathrm{total}}}=\frac{M_{\mathrm{PBR}}+M_{\mathrm{drain}}-M_{0}}{t\cdot (V_{\mathrm{PBR}}+\sum_{i=1}^{n}V_{i})}=\frac{k\cdot \left(\mathrm{OD}750_{f}\cdot V_{\mathrm{PBR}}+\sum_{i=1}^{n}\mathrm{OD}750_{i}\cdot V_{i}-\mathrm{OD}750_{0}\cdot V_{\mathrm{PBR}}\right)}{t\cdot (V_{\mathrm{PBR}}+\sum_{i=1}^{n}V_{i})}$$
  where M_total_ is the total biomass accumulated and drained from a PBR harvested during time *t*, including the biomass accumulated in a PBR at the end of the cultivation and the total biomass drained from a PBR during the semi-continuous mode; M_0_ is the inoculate biomass; V_total_ is the whole volume gained from the cultivation cycle, including the total volume drained from the PBR in a semi-continuous mode ($\overset{n}{\underset{i=1}{\sum }}V_{i}$) and the final volume in the PBR (V_PBR_); OD750_f_ is the optical density of the culture at 750 nm, measured at the end of the cultivation; OD750_i_ and V_i_ are the optical densities at 750 nm and the volumes of suspension periodically drained from the PBR in the semi-continuous mode; n is the number of drains; OD750_0_ is the optical density at 750 nm, measured at the beginning of cultivation.
- The specific growth rate (μ) was estimated by the change in the culture OD (h^−1^):
  (5) $$\mu =\ln\left(\mathrm{OD}750_{2}/\mathrm{OD}750_{1}\right)/\left(t_{2}-t_{1}\right)$$
  where OD750_1_ и OD750_2_ are the optical densities of the culture at 750 nm, measured during time 1 (t_1_) and time 2 (t_2_), respectively.
- The biomass doubling time (T_dbl_) was also calculated from the specific growth rate:
  (6) $$T_{\mathrm{dbl}}=\ln\left(2\right)/\mu$$

The final productivity (P_f__in_) of the strains was determined by the concentration of biomass at the end of the cultivation.

### 2.6. Statistics

Every measurement was performed in three or more technical repetitions. The graphs of growth curves represent the actual values obtained in the individual experiments. The mean values for the replicates and their standard deviations are mentioned in the text.

## 3. Results

### 3.1. Conversion of Optical Density to Dry Biomass Productivity

**Figure 3:**
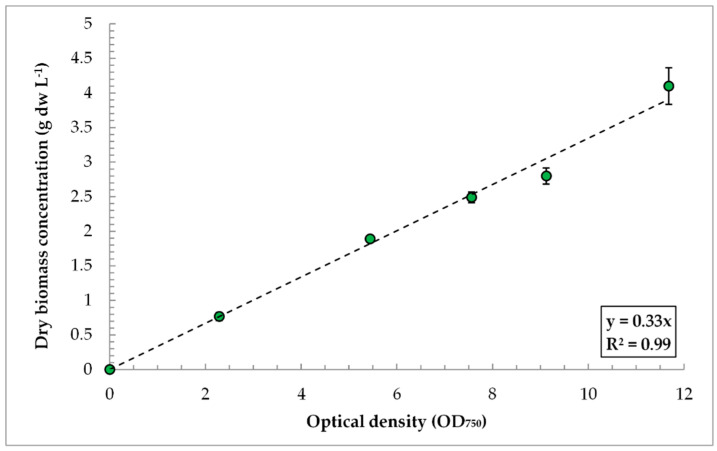
Values of dry biomass productivity at different values of optical density for *C. sorokiniana* IPPAS C-1. Mean ± SD (N = 3).

The coefficient of conversion of OD_750_ into absolute values of dry biomass weight per liter (g dw L^−1^) of suspension was estimated. Figure 3 shows the values of the biomass concentration of *C. sorokiniana* IPPAS C-1 corresponding to the various OD_750_ of the culture. The multiplier for converting the OD values into the value of biomass concentration corresponds to the slope of the linear trend line and is equal to 0.33. This conversion coefficient can be applied to all cases of *C. sorokiniana* IPPAS C-1 cultivation when measuring its OD_750_, regardless of the volumes and characteristics of the PBRs. In this work, the indicators of the PBRs’ productivity and efficiency were determined using the conversion coefficient k.

### 3.2. Batch Cultivation Mode

**Figure 4:**
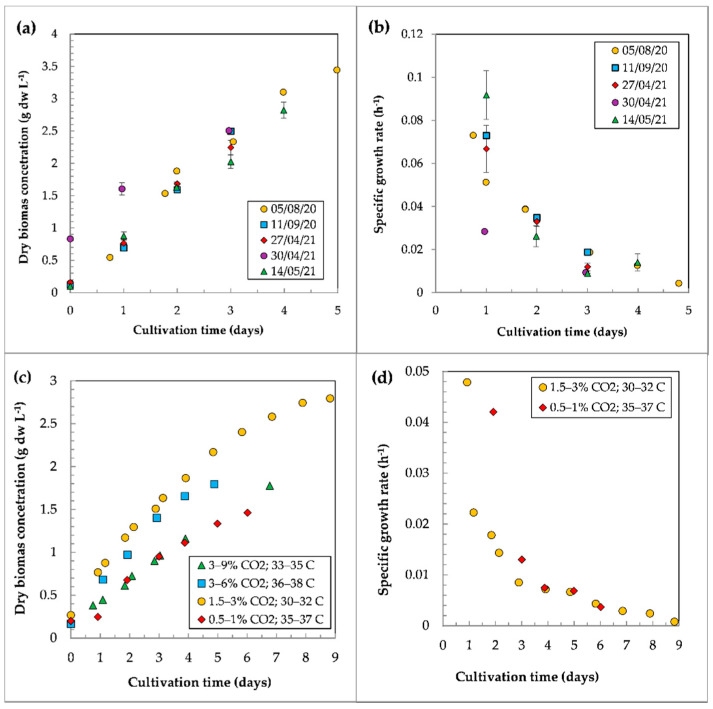
Growth characteristics of *C. sorokiniana* IPPAS C-1 in a batch mode. (**a**) PBRs FPH-5: growth curves, and (**b**) specific growth rates (h^−1^). Mean ± SD (N = 3). (**c**) PBR FPV-70: growth curves, and (**d**) specific growth rates (h^−1^) (individual data points).

The growth characteristics of the batch cultures of *C. sorokiniana* IPPAS C-1 are presented in Figure 4 and Table S1. The experiments were carried out as follows:

- Simultaneous batch cultivation in two identical PBRs FPH-5 at 30–32 °C under the continuous average irradiation of 500 μmol photons m^−2^ s^−1^ and the CO_2_ concentration in the GAM of 1.5–2% (Figure 4a,b).
- Batch cultivation in PBR FPV-70 under the continuous average irradiation of 143 μmol photons m^−2^ s^−1^ at various CO_2_ concentrations and temperatures (Figure 4c,d).

In PBRs FPH-5, the cultures were at the linear growth phase during the time of the experiments (Figure 4a). At day 3, ρ reached 2.2 ± 0.3 g dw L^−1^. At day 5, ρ reached 3.4 ± 0.07 g dw L^−1^. The maximum µ achieved in the batch mode was 0.103 h^−1^. At day 5, the µ values were close to zero, and further cultivation was ineffective (Figure 4b). Notably, when the initial ρ was higher (0.80 and 0.86 g dw L^−1^), the maximum µ values were 3–4 times lower compared to the runs with a lower initial concentration (0.08 and 0.12 g dw L^−1^). In the batch mode, the maximum P_sp_ was 0.82 g dw L^−1^ d^−1^, as calculated from the first three days of cultivation (Table S1).

In PBR FPV-70, the growth of *C. sorokiniana* IPPAS C-1 in the batch mode was significantly affected by CO_2_ concentration and temperature under the constant irradiation of 143 μmol photons m^−2^ s^−1^. The highest ρ = 2.8 g dw L^−1^ was achieved at 30–32 °C with 1.5–3% CO_2_ supply in 9 days (Figure 4c). Similar growth curves were observed at 36–38 °C and 3–6% CO_2_ during first 4 days. However, on day 5, the ρ values dropped by 17%. Cultures grown at 35–37 °C and 0.5–1% CO_2_ for 6 days displayed the lowest growth rates and the lowest P_fin_ of 1.26 g dw L^−1^. The specific growth rates for the cultures with maximal and minimal ρ values are shown in Figure 4d.

### 3.3. Semi-Continuous Cultivation Mode

**Figure 5:**
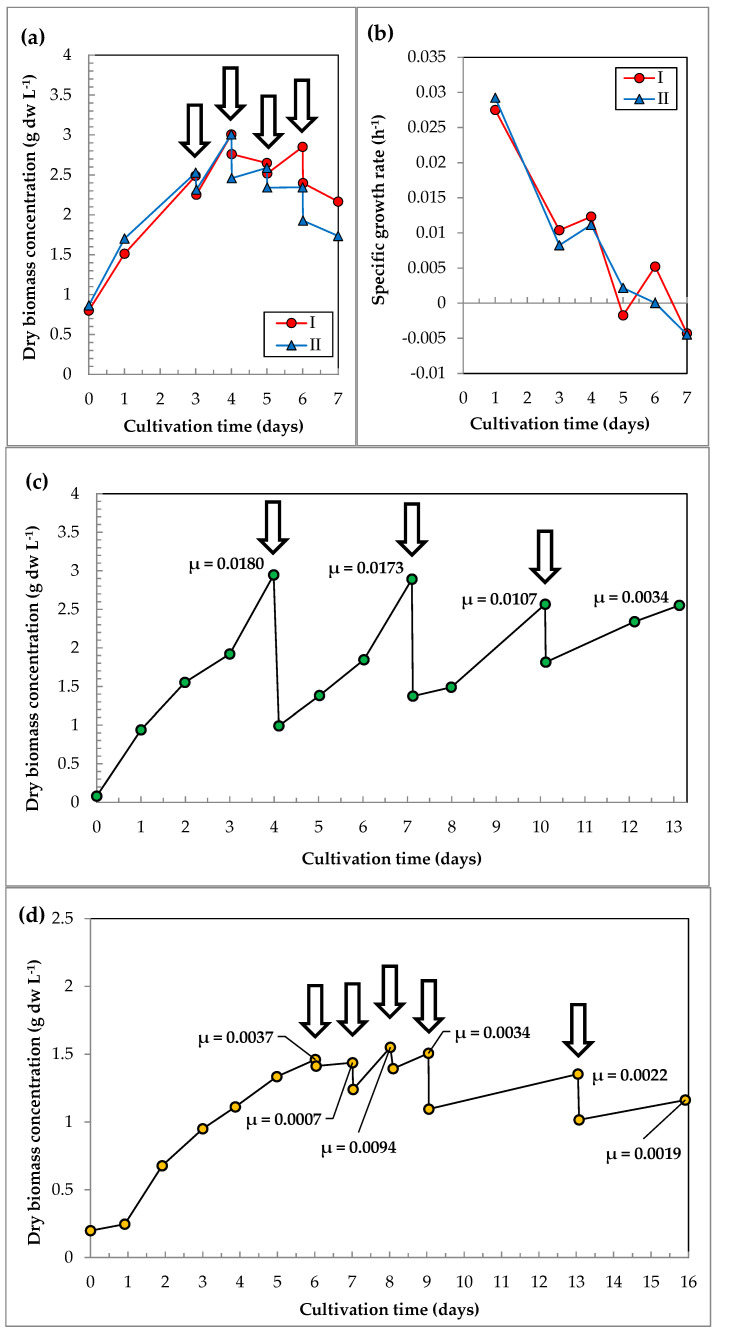
Growth characteristics of *C. sorokiniana* IPPAS C-1 grown in semi-continuous and combined modes. PBRs FPH-5 I and II, daily dilutions: (**a**) growth curves; (**b**) specific growth rates (h^−1^). (**c**) PBR FPH-5 II, 3-day periodic dilutions: growth curve and specific growth rates. (**d**) PBR FPV-70 in a combined mode: growth curve and specific growth rates. Arrows indicate the time of suspension withdrawal and dilution with the fresh growth medium. The individual data points are presented. µ—the specific growth rate.

Figure 5 demonstrates the growth characteristics of *C. sorokiniana* in the semi-continuous and combined modes of cultivation in PBRs, and the main results are presented in Table S2. The experiments were carried out in the following variants:

- Semi-continuous cultivation at 30–32 °C under the continuous average irradiation of 500 μmol photons m^−2^ s^−1^ and 1.5–2% CO_2_ in two identical PBRs FPH-5. From day 3 of the cultivation, the daily draining of 700 mL of the suspension (four drains) and dilution with a nutrient medium (Figure 5a,b) were performed.
- Semi-continuous cultivation in one PBR FPH-5 under the conditions described above. After day 4, 3.6 L of the suspension was withdrawn (three drains), and the PBR was refilled with the fresh nutrient medium. The withdrawal and refilling were repeated every 3 days (Figure 5c).
- Combined mode in PBR FPV-70 consisting of a 6-day batch mode and then a 3-day semi-continuous mode of cultivation with daily draining of 7 L and refilling (dilution) with the fresh nutrient medium (four drains), followed by the draining of 20 L with dilution periods of 4 and 3 days (two drains). The total cultivation time was 16 days (Figure 5d) at 35–37 °C, a continuous average irradiation of 143 μmol photons m^−2^ s^−1^, and a 0.5–1% CO_2_ supply.

The cultivation employing the semi-continuous mode in PBRs FPH-5 gained, in total, 2.8 L of the suspension, with an average ρ of 2.6 g dw L^−1^. The maximum increment of biomass concentration was observed after the first dilution; then, the growth became unstable, and after the next dilutions, the biomass concentration gradually decreased in both experimental variants (Figure 5a). After 3 days of cumulative growth, the cultivation was changed to the semi-continuous mode; an increase in the specific growth rate after the first dilution was followed by a gradual decrease (Figure 5b). The average total yield after 1 week of cultivation for 3 days in the batch mode and 4 days in the semi-continuous mode was 16.9 g dw from 8 L of the suspension. The average P_sp_ was at the level of 0.3 g dw L^−1^ d^−1^.

In other rounds of experiments, when the 4-day accumulative (batch) cultivation was changed to the semi-continuous mode, the ρ values were at the range of 2.5–3.0 g dw L^−1^ (Figure 5c). At the end of each 3-day cycle, 3.6 L of the suspension was drained. In total, after three cycles, 10.8 L of the suspension was drained, with an average ρ of 2.9 g dw L^−1^. The total yield for 13 days of cultivation (4 days of the batch mode and 9 days of the semi-continuous mode) was 44 g dw from 15.4 L of the suspension. Accordingly, the average productivity resulted in 0.2 g dw L^−1^ d^−1^.

Switching to a daily drain-dilution cycle in the PBR FPV-70, when 7 L portions of the culture were withdrawn, the ρ values were maintained at 1.22–1.62 g dw L^−1^. Thus, in total, 21 L were drained, with an average ρ = 1.6 g dw L^−1^.

Further 3–4-day cycles resulted in a decrease in the OD_750_ values by 10% and 14%, respectively. As a result, after continuous cultivation, the ρ in the suspension dropped from 1.52 to 1.16 g dw L^−1^. Draining of 20 L of the suspension was carried out twice: first after 4 days and then after 3 days of cultivation. Accordingly, the first drain with a ρ of 1.56 g dw L^−1^ yielded 31 g dw. The second drain with a ρ of 1.42 g dw L^−1^ yielded 28.3 g dw. Over the entire cultivation cycle of 13 days, the total yield was estimated as 141 g dw.

### 3.4. Accumulation of C. sorokiniana Biomass during the Cultivation Cycle: From the Laboratory to the Pilot Scale

**Figure 6:**
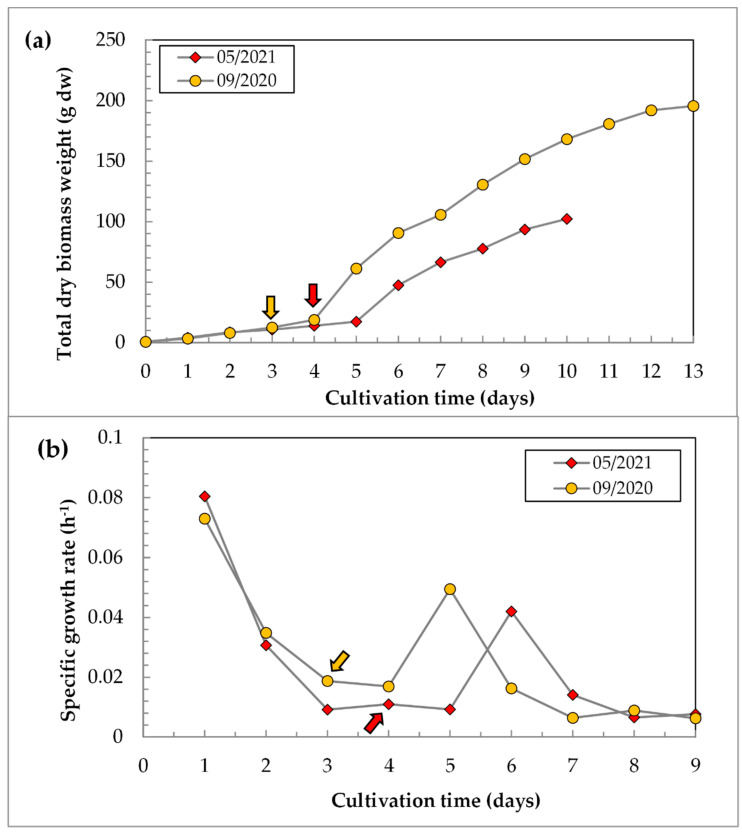
Growth characteristics of *C. sorokiniana* IPPAS C-1 in the batch mode, starting from the inoculation of the PBR FPH-5 until the end of the batch cultivation in the PBR FPV-70: (**a**)—accumulation of biomass in absolute values of dw; (**b**)—specific growth rate. Colored arrows represent the time of suspension transition from PBR FPH-5 to PBR FPV-70. Individual data points. Growth conditions are represented in Table S1 for the replicates (exp.code): 11/09/20–14/09/20 for orange data points and 14/05/21 II–18/05/21 for red data points. Average irradiation for PBR FPH-5—500 μmol photons m^−2^ s^−1^; for PBR FPV-70—143 μmol photons m^−2^ s^−1^.

The accumulation of dry biomass of *C. sorokiniana* during two different cultivation cycles is presented in Figure 6. The starting point of each replicate corresponds to the amount of dry biomass accumulated in 250 mL of the culture grown in a glass vessel in a laboratory cultivation system, which was poured into PBR FPH-5. These replicates have differences in growth conditions (see the Figure 6 caption and Table S1) and in the duration of the accumulation period in PBR FPH-5. The case 05/2021 has a lower final yield in the dry biomass: after 4 days of cultivation in PBR FPH-5, 14 g dw was accumulated. This biomass was transferred into PBR FPV-70, and after 6 days of cultivation, 102 g dw was obtained. In total, the described 10-day cycle of cultivation resulted in a 167× increase in biomass (g dw). In the replicate 09/2020, where the transfer from PBR FPH-5 to PBR FPV-70 was on the third day of cultivation, the final results were higher: 12.5 g dw in PBR FPH-5 and 168.2 g dw in PBR FPV-70 were accumulated in 10 days. The total duration of the cultivation in this replicate lasted for 13 days. The final specific productivity of the culture was P_sp_ = 0.22 g dw L^−1^ d^−1^, with a 327× increase in biomass (from 0.6 to 196 g dw in 13 days).

## 4. Discussion

### 4.1. Characteristics of the Flat-Panel Photobioreactors Tested in This Study

The parameters, such as the average irradiation (E_e_) and the ratio of the illuminated surface to the volume (SA/V), reflect the light characteristics of PBRs. An increase in these values with a corresponding increase in CO_2_ supply leads to a noticeable increase in the specific productivity of the biomass (P_sp_) [9,17]. However, the limitations may be associated with the additional costs for light sources and light-penetrating surfaces, power consumption, the minimization of dark volumes, etc. In addition, the growth of some algal strains may be inhibited by high-light intensities. The important indicator of PBR is the specific power consumption (W_sp_), which proportionally grows with an increase in irradiation. It shows the amount of electrical energy expended to power the light sources and the other electrical devices of the system per liter of cultivated suspension per unit of time. This parameter can be used to estimate the energy costs of the mass production of microalgae in the considered types of a PBR.

The efficiency and productivity of different types of PBRs are estimated using various criteria. These parameters reflect the constructive and thermophysical characteristics of the PBRs [9,10,11,12,13,14,15,17,18,19,21,22,23]. The key parameters of the PBRs tested in this work are presented in Table S3.

It is known that the production of 1 g dw of microalgae biomass requires about 1 L of CO_2_ [7,14,15,24,25,26]. The ratio M/V_CO2_ reflects the efficiency of the total CO_2_ conversion into biomass and can be related to both the ventilation coefficient, i.e., the GAM consumption per liter of suspension (K_vent_), and the design of PBR [25,26]. This ratio also reflects the congruence of all cultivation parameters (including PBR characteristics) to the optimal conditions for culture growth.

Here, the P_sp_ value was two times higher in PBR FPH-5 than in PBR FPV-70. At the same time, 1 L of the suspension in PBR FPH-5 was illuminated 3× more intensively in terms of specific power consumption (W_sp_), and it received 6.4× more CO_2_ (V’_CO2_) compared to PBR FPV-70.

The scaling up of the cultivation process inevitably leads to an increase in the absolute costs of PBR operations under optimal conditions. The task of scaling up is to maintain the optimal specific characteristics of a PBR: the ratio of the illuminated surface to the volume; the electricity consumption per liter of suspension; the CO_2_ to biomass conversion ratio for the selected period of time; the values of irradiation on the active surfaces of a PBR, etc. Table S3 demonstrates that the specific indicators for irradiation, electricity consumption, and the ventilation coefficient are three times lower, and the specific productivity of the biomass was two times lower in PBR FPV-70 than in FPH-5. The absolute values of the consumed electric energy (W_sp_·V), however, were three times higher in PBR FPV-70, evidently, due to the 14× difference in the reactor volumes. For comparison, the power consumption of 25-L PBR FPV [24] was~ 6.8 W L^−1^, which was mostly consumed by the lighting system, similarly to the PBRs described here. Another study [22] presented PBRs with working volumes of 8 and 90 L consuming electricity at 28–40 W L^−1^ and 15 W L^−1^, respectively.

In our study, irradiation in both types of reactors appeared insufficient at ρ > 0.66 g dw L^−1^. It was reported that the optimal light intensity close to the surface in the photic layer is in a range of 200–400 μmol photons m^−2^ s^−1^ [9]. These values were shown to be optimal for most studied strains during their intensive growth. Achieving such irradiation levels on the cell surface requires much higher irradiation on the light-receiving surfaces. According to the data cited above, the maximum permissible irradiation level of the cell surface is 2000 μmol photons m^−2^ s^−1^. Low irradiation leads to shorter light paths and the appearance of dark volumes, in which cell respiration significantly reduces the productivity [11,12].

The optimal SA/V ratio is defined in the range of 43–73 for different types of PBRs, including FPV [17]. Higher values of the SA/V ratio can lead to photoinhibition and the overheating of a culture. Lower values may cause an increase in dark volumes. The SA/V ratios of our PBRs are close to optimal.

It is possible to intensify the absorption of light quanta by cells using intensive mixing, which provides the access of each cell to the light-receiving surface of a reactor. The favorable light exposure can be obtained by a combination of the optimal illumination with the intensive mixing [9]. In the case of GAM bubbling, the intensity of the mixing is determined by K_vent_, which reflects the conditions for optimal suspension mixing and the supply of cells with CO_2_. The inconsistency of the bubbling intensity with the intensity of CO_2_ assimilation can lead to the emergence of a CO_2_ concentration gradient in the pericellular zones. This may result in serious errors in the estimation of the growth’s dependence on the CO_2_ supply. However, a K_vent_ of 300 L GAM h^−1^, selected as the optimal value in some publications, seems to be extremely high for the operation in pilot-scale volumes. While the K_vent_ of our PBR FPV-70 was almost three times lower than that of PBR FPH-5, the M/V_CO2_ ratio was almost two times higher (Table S3). This phenomenon can be explained, first of all, by the geometry of the PBRs. In PBR FPH-5, the light-receiving surface coincides with the evaporation surface, and the light path is equal to the shortest path of a GAM bubble. In PBR FPV-70, these surfaces do not coincide: the bubble path in PBR FPV-70 is much longer than that in PBR FPH-5. It is known that a longer bubble path (or residence time of gas bubbles) allows for higher CO_2_ assimilation [27].

### 4.2. Batch Cultivation Mode

Several factors may limit the growth rate of the culture. The ρ values increased in a linear phase (Figure 4a,b); however, the µ values noticeably decreased after two days of cultivation (Figure 4c,d). The cultures had no limitations in the supply of CO_2_ or nutrients. Therefore, insufficient light intensity could limit the growth rate.

Earlier work [24] described the cultivation of *C. pyrenoidosa* IPPAS C-2 in PBRs of various geometries, including a flat-panel vertical 25 L PBR. The initial biomass concentration was 0.24 ± 0.07 g dw L^−1^, and it reached 1.84 ± 0.11 g dw L^−1^ in 8 days of cultivation. This value corresponds to a P_sp_ of 0.23 g dw L^−1^ d^−1^. Similar P_sp_ values (up to 0.20 g dw L^−1^ d^−1^) were obtained during the cultivation of *C. sorokiniana* in the flat-panel vertical 100–300 L PBRs [17]. These values fall within the range of our results obtained in FPV-70, and they are three times lower than the values obtained in FPH-5.

The cultivation of *Chlorella* sp. NTCU-2 in a closed 4 L cylindrical PBR under increased light intensity (up to 300 μmol photons m^−2^ s^−1^) and CO_2_ concentration (up to 5%) resulted in a specific productivity of 0.9 g dw L^−1^ d^−1^ at 26 °C [18]. These values are slightly higher than those obtained in PBR FPH-5, and this is probably due to the lower light path that increased the average light irradiation level on the cell surface [14,15]. The higher amount of CO_2_ fed into the PBR, together with the more effective aeration, may be another reason for the increased productivity values.

Apart from the constructive features of various PBRs and variations in cultivation regimes, the genetic, biochemical, and biophysical properties of a particular algal strain may be critical for the final productivity. In order to improve the photosynthetic efficiency, the light penetration within a culture, and the light-to-biomass conversion rate, many efforts have been made to reduce the antenna size of photosystem II (PSII) in several algal species. Truncated antenna mutants have been generated either by random UV [28] or chemical [29,30] mutagenesis or by more delicate genetic engineering approaches [31,32]. The reduction of the PSII antenna size in *Chlorella vulgaris* (in particular, by the suppression of chlorophyll *b* biosynthesis) resulted in substantially higher photosynthetic rates and an increase in biomass productivity (from 0,43 to 0,56 g dw L^−1^ day^−1^) at a high irradiance (>200 μmol photons m^−2^ s^−1^) [30]. In a model of *Chlamydomonas reinhardtii*, however, the antenna truncation had no significant impact on productivity (~2 g dw m^−2^ h^−1^) [33]. At the same time, the reductions in the light-harvesting antenna size increased the susceptibility to photodamage [31], which can be overcome by the increase in the carotenoids content [30].

The enhanced illumination, CO_2_ fixation rate, and economical water supply may be provided by the alternative cultivation strategy, using algal cells immobilized to a substrate [34]. This approach, however, requires scaling up to meet the requirement of the mass culture industry.

### 4.3. Semi-Continuous Cultivation Mode

The semi-continuous cultivation in PBR FPH-5 displayed lower values of P’_sp_ compared to batch cultures. Daily dilutions in 3-day cycles gained up to 0.3 g, whereas batch cultivation gave twice more, on average (0.64 g dw L^−1^ d^−1^). At the same time, 3-day cycles allowed for the maintenance of µ > 0 for a longer time than the 1-day cycle and, finally, provided a higher total yield. The values of ΔM_ave_ were similar to the batch mode. Periodic renewals of the nutrient medium, which diminish concentrations of the accumulated metabolites, may be preferable for industrial biomass production. In addition, draining 72% of the suspension and diluting it with a fresh medium allows more efficient light absorption, which leads to an immediate increase in the µ value. For comparison, the P’_sp_ values obtained for *Chlorella* sp. NTCU-2 cultivated in a cylindrical closed 4 L PBR in the semi-continuous mode were 0.6, 0.5, and 0.5 g dw L^−1^ d^−1^ for 2-, 3-, and 8-day cycles, respectively [18]. Showing a similar tendency to decrease with the time of cultivation, these P’_sp_ values exceed almost twice the values obtained in the present study using *C. sorokiniana* IPPAS C-1. In our work, this may be due to the lower irradiation level used during the experiments.

Qualitatively, in PBR FPV-70, the growth tendency did not differ from that of PBR FPH-5. The differences appeared only in the absolute values of ρ and µ due to a significant difference in volume and light irradiation (Figure 5d).

Notably, in the semi-continuous mode, µ constantly decreased with the time of cultivation (Figure 5c). Such tendency was observed in the subsequently diluted *C. vulgaris* MACC-1 and is explained by the varying consumption rates for different minerals, which ultimately lead to an imbalance in the nutrient medium after several dilutions [23]. This may also be affected by the aging of the culture, cell precipitation, foaming, the contamination of lighting modules, etc. [9].

### 4.4. Accumulation of C. sorokiniana Biomass during the Cultivation Cycle: From the Laboratory to the Pilot Scale

During the cultivation of *C. sorokiniana* IPPAS C-1, the maximum µ was observed on the first day of cultivation in PBR FPH-5 for both variants (Figure 6b). The first day of cultivation in PBR FPV-70 revealed a noticeable lag phase (Figure 6a). Supposedly, the lag phase was caused by significant changes in the cultivation conditions after the transition from PBR FPH-5 to PBR FPV-70. The increase in the µ in the PBR FPV-70 in all variants (Figure 6b) can be explained by a significant decrease in ρ after the dilution with a fresh medium and the successful adaptation of the culture to new conditions.

The difference in the absolute values of the growth characteristics between the presented cases could be explained by the maintenance of similar cultivation conditions after the transfer from one volume to another in variant 09/2020 and the significant change in the CO_2_ concentration and the temperature of the cultivation in variant 05/2021 after the culture transfer. A drop in the concentration of CO_2_ from 1.5–3% to 0.5–1% and a rise in the temperature from 31–35 to 35–37 °C might be the main reasons for the decrease in the specific productivity. Finally, variant 09/2020 had a 1.7× higher P_sp_ than variant 05/2021 in 10 days of cultivation (0.24 g compared to 0.14 g dw L^−1^ d^−1^, respectively).

## 5. Conclusions

A set of experiments on *Chlorella sorokiniana* cultivation under various conditions were carried out in the newly developed semi-closed type PBRs. The coefficient of OD_750_ conversion to the dry biomass concentration was empirically determined to be 0.33. The results of cultivation cycles from a laboratory volume (0.25 L) to a pilot volume (70 L) are presented. The key parameters, schemes, and principles of the PBRs’ operation are described.

Overall, the batch growth gives higher values of specific parameters, and it is preferable for achieving high biomass yields in short-term (up to one week) cultivation. The semi-continuous mode is better for long-term cultivation because of the periodical draining and refreshing of the cell populations in a biomass. An increase in biomass by 367 times (from 0.6 to 196 g dw) was achieved in 13 days of cultivation, with an average specific productivity of 0.21 ± 0.06 g dw L^−1^ d^−1^.

## Data Availability

Not applicable.

## Supplementary Materials

The following are available online at https://www.mdpi.com/article/10.3390/life12091309/s1, Figure S1: The laboratory cultivation system, which holds 250 mL glass vessels. Table S1: Experimental results from batch mode cultivation. Table S2: Parameters of the semi-continuous mode cultivation in the PBRs FPH-5 and FVP-70. Table S3: Key parameters and criteria for evaluating the efficiency and productivity of PBRs calculated for three days of cultivation in the batch mode. Video S1: The laboratory cultivation system, which holds 250 mL glass vessels. Video S2: The operation of the PBR FPH-5. Video S3: The operation of the PBR FPV-70.

## Abbreviations

The following abbreviations are used in this manuscript:

µ	specific growth rate
g dw	gram of dry weight
GAM	gas-air mixture
IPP RAS	Institute of Plants Physiology of Russian Academy of Sciences
k	conversion coefficient between optical density measured at 750 nm and dry biomass concentration
K_vent_	ventilation coefficient
LED	light-emitting diode
LSIC	laboratory system for intensive cultivation
ρ	biomass concentration
M_0_	starting weight of dry biomass in the PBR after inoculation
M_drain_	total weight of dry biomass drained from the PBR during the total time of semi-continuous cultivation
M_total_	total weight of dry biomass accumulated, harvested, and drained from the PBR during the total time of cultivation
M_PBR_	weight of dry biomass in the working volume of the PBR at the moment
OD_750_ (or OD750)	optical density at 750 nm
P’_sp_	specific productivity for the semi-continuous mode
PBR	photobioreactor
PBR FPH	flat-panel horizontal photobioreactor
PBR FPV	flat-panel vertical photobioreactor
P_fin_	final productivity
P_sp_	specific productivity
T_dbl_	biomass doubling time
t_total_	total time (duration) of the cultivation
TES	trace element solution
V_total_	total volume of accumulated, harvested, and drained culture during the total time of cultivation
V_i_	volume of culture drained from the PBR during a single drain in semi-continuous cultivation
V_PBR_	working volume of PBR
