# Supplementary material for: Cultivation of Chlorella sorokiniana IPPAS C-1 in Flat-Panel Photobioreactors: From a Laboratory to a Pilot Scale
Source: Life (Basel). 2022 Aug 25;12(9):1309. doi: 10.3390/life12091309 (PMC9506280; doi:10.3390/life12091309)
Supplement: Supplementary file 1 [file life-12-01309-s001.zip › TablesS1,S2,S3.pdf]

Table S1. Experimental results from batch mode cultivation in PBRs FPH-5 and FPV70 (individual data points).

PBR	Exp. code (starting date) *	Conditions n_{CO2} / Temp. (% / °C)	Starting dry biomass conc. (g dw L^{-1})	P_{sp} total time (g dw L^{-1} d^{-1})	P_{sp} in 3 days (g dw L^{-1} d^{-1})	P_{fin} (g dw L^{-1})	μ_{max} (h^{-1})	M_{total} (g dw)	t_{total} (d)	ΔM_{ave} ** (g dw d^{-1})
FPH-5	05/08/20	1.5-2 / 30-32	0.15	0.66	0.72	3.31	0.067	16.5	5	3.30
	11/09/20	1.5-3 / 27-30	0.12	0.82	0.82	2.50	0.073	12.5	3	3.97
	27/04/21 I	1.0-1.8 / 28-29	0.19	0.65	0.65	1.94	0.056	9.7	3	3.23
	27/04/21 II	1.0-1.8 / 28-30	0.13	0.74	0.74	2.23	0.078	11.2	3	3.73
	30/04/21 I	1.2-1.8 / 29-31	0.80	0.56	0.56	1.69	0.027	8.4	3	2.80
	30/04/21 II	1.2-1.8 / 30-32	0.86	0.55	0.55	1.66	0.029	8.3	3	2.77
	14/05/21 I	1.5-2 / 31-35	0.12	0.64	0.67	2.58	0.080	12.9	4	3.23
	14/05/21 II	1.5-2 / 31-35	0.08	0.72	0.61	2.87	0.103	14.3	4	3.60
FPV-70	10/08/20	3-9 / 33-35	0.18	0.24	0.30	1.60	0.042	111.9	7	15.98
	27/08/20	3-6 / 36-38	0.17	0.33	0.42	1.63	0.054	114.1	5	22.82
	14/09/20	1.5-3 / 30-32	0.27	0.29	0.44	2.53	0.048	176.9	9	19.66
	18/05/21	0.5-1 / 35-37	0.20	0.21	0.31	1.26	0.042	88.4	6	14.73

* I and II are the numbers of independent biological repetition.

** ΔM_{ave} is an average biomass yield per a day of cultivation calculated as full biomass (M_{total}) divided by duration of cultivation (t_{total}).

Table S2. Parameters of semi-continuous mode cultivation in PBRs FPH-5 and FVP-70.

PBR	Period of dilution (d)	Num. of drains	Starting conc. of culture* (g dw L^{-1})	Drained culture per period Volume / Part (L / %)	μ_{max} (h^{-1})	Ave. yield per drain (g dw)	Average P'_{sp} per period (g dw L^{-1} d^{-1})	Total yield ** (g dw)	Total volume drained** (L)	Total duration ** (d)	ΔM_{ave} (g dw d^{-1})	Average P'_{sp} full time (g dw L^{-1} d^{-1})
FPH-5	1	4	2.37±0.11	0.7 / 13.4	0.012	1.78 ±0.15	0.307 ±0.063	16.9±0.9	8	7	2.41	0.301
	3	3	1.39±0.28	3.6 / 72	0.017	10.43 ±0.54	0.426 ±0.138	44.0	15.4	13	3.38	0.220
FPV-70	1	3	1.38±0.07	7 / 10	0.009	10.87 ±0.47	0.145 ±0.083	138.2	88.75	9	15.36	0.173
	3-4	2	1.06±0.04	20 / 28.6	0.002	29.70 ±1.45	0.058 ±0.007	140.6	108.5	13	8.35	0.100

* Starting concentration of culture is the value of culture concentration in PBR at the beginning of semi-continuous mode and is maintained by draining and dilution. Mean±SD (*N* = number of drains).

** Total yield, total volume drained and total duration consider full time of cultivation beginning from batch mode to achieve the starting culture concentration and finishing by draining of whole culture volume.

Table S3. Key parameters and criteria for evaluating the efficiency and productivity of PBRs calculated for three days of cultivation in batch mode

PBR	Average irradiation Ee , μmol photons m^{-2} s^{-1}	The ratio of the illuminated surface to the volume SA/V, m^2 m^{-3}	Specific power consumption* Wsp , W L^{-1}	Maximum specific productivity of biomass Psp , g dw L^{-1}·d^{-1}
FPH-5	500	48	21.6	0.89±0.05
FPV-70	143	35.79	6.86	0.43±0.05
**PBR**	**Ventilation coefficient, Kvent, L.GAM L^{-1} h^{-1}**	**Specific consumption of CO_2 V'CO_2 , L.CO_2 L^{-1} d^{-1}**	**Accumulated biomass M, g dw**	**CO_2 to biomass conversion ratio M / VCO_2 , g dw (L.CO_2)$^{-1}$**
FPH-5	24.07	9.68±1.18	14.05±1.71	0.07
FPV-70	8.57	1.52±0.21	87.62±12.09	0.14

* - electric power consumption of the PBRs used for light system and ventilation of GAM;
