# Supplementary figures and images for: Cultivation of Chlorella sorokiniana IPPAS C-1 in Flat-Panel Photobioreactors: From a Laboratory to a Pilot Scale
Source: Life (Basel). 2022 Aug 25;12(9):1309. doi: 10.3390/life12091309 (PMC9506280; doi:10.3390/life12091309)

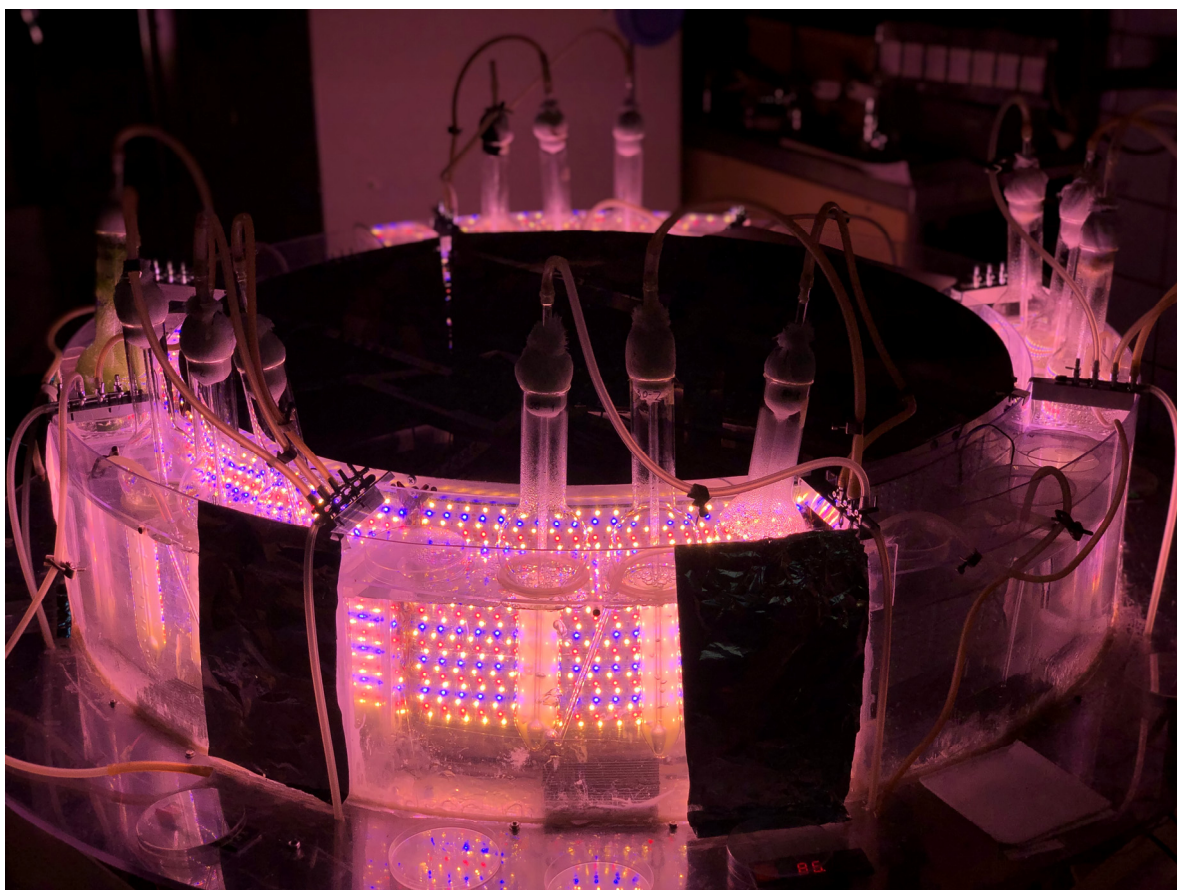

**Figure S1.** The laboratory cultivation system, which holds 250 mL glass vessels.

Supplement: Supplementary file 1 [file life-12-01309-s001.zip › Figure S1.pdf]
